# CT-guided percutaneous laser ablation of metastatic lung cancer: three cases report and literature review

**DOI:** 10.18632/oncotarget.13901

**Published:** 2016-12-10

**Authors:** Qiyu Zhao, Guo Tian, Fen Chen, Liyun Zhong, Tian’an Jiang

**Affiliations:** ^1^ Department of Ultrasonography, First Affiliated Hospital, Zhejiang University School of Medicine, Hangzhou China; ^2^ State Key Laboratory for Diagnosis and Treatment of Infectious Diseases, Collaborative Innovation Center for Diagnosis and Treatment of Infectious Diseases, The First Affiliated Hospital, Zhejiang University School of Medicine, Hangzhou, China; ^3^ Department of Hepatobiliary Pancreatic Surgery, First Affiliated Hospital, Zhejiang University School of Medicine, Hangzhou, China

**Keywords:** laser ablation, CT-guided, computed tomography, lung, lung cancer

## Abstract

**Objective:**

To report the efficacy and safety of CT-guided percutaneous laser ablation (PLA) for metastatic lung tumors.

**Methods:**

Three cases of metastatic lung cancer underwent CT-guided PLA, and we searched for previously published articles on the minimally invasive CT-guided RFA or MWA for lung tumors in recent five years.

**Results:**

With the guidance of CT, all lesions had good prognosis under laser ablation. Case 1 suffering from severe pulmonary dysfunction and diffuse pulmonary bullae, had small pneumothorax. CT scan obtained four months following the ablation showed two lesions had complete responses and one partial response. Case 2 had successful complete response with absent lung mass, and also had a good postoperative condition without any discomfort in the two-month follow-up. Case 3 showed partial response and improved greatly after five months. 962 cases (mean age of 45.7 years, 62.2% male) of 1297 lung tumors with detailed information were identified from 27 articles. Of these cases, the minority manifested complications such as pneumothorax, hemoptysis, hemothorax, pneumonia, pain and fever.

**Conclusions:**

Percutaneous CT-guided PLA could be a safe and promising minimally invasive treatment for patients with primary lung cancer or unresectable pulmonary metastases, especially multineedle PLA in large tumors, which still needs more large-scale prospective studies to convince this method in the future.

## INTRODUCTION

Lung cancer is the most common cause of cancer-related death in the world. It was reported that 226,160 new patients of lung cancer was diagnosed in 2012, and it was evaluated that 160,340 deaths were related to lung cancer [1]. Smoking is the most dangerous factor for lung cancer. Environmental and occupational exposures to carcinogens also have important impacts on this disease. Meanwhile, lung is the second most common site of metastases for colon cancer after liver and traditionally, surgery is the main treatment option. However, it is more expensive and has higher complication risks such as atrial fibrillation, prolonged air leaks, myocardial infarction, recurrent nerve injury, bleeding, pneumonia, and bronchial stenosis [2, 3]. About 10% to 15% patients who had a surgical resection for colorectal cancer may develop lung metastasis [4]. In recent years, emerging studies have displayed that CT-guided RFA and MWA are feasible for patients with primary lung cancer or unresectable pulmonary metastases, in which pneumothorax, hemoptysis, pneumonia, pleural effusion may occur [5-22]. It was reported that the 3-year overall survival rate following RFA for pulmonary metastases was nearly 50% against that (60%) of patients treated with pulmonary metastasectomy [4]. However, RFA have a higher impedance in the lung than in the liver, which means poor energy was spread due to high impedance and charing, thus it is difficult to identify whether the lesions are completely ablated or not.

Additionally, percutaneous laser ablation (PLA) is minimally invasive by introducing the position of laser catheters into the lesions, followed by thermal ablation of targeted tumors through laser energy. PLA has more advantages than other ablative methods including the lower risk of bleeding, infection or injuries of adjacent organs [23], which enable PLA to be valuable for the treatment of small tumors adjacent to vital but subtle regions.

Recently, PLA has been increasingly applied in the treatment of benign and malignant diseases in the liver and thyroid through the guidance of ultrasonography (US) as well as Magnetic Resonance Imaging (MRI) [24-28]. In particular, CT-guided PLA showed a safe, minimally invasive and promising therapy in some lesions such as adrenal metastases and osteoid osteomas [23, 29]. In previous study, percutaneous CT-guided PLA of lung tumors presented complete ablation of lung metastases and lung cancers with the average energy of 46 kJ (18-102 kJ) through 9F trocar, of which pneumothorax, endothelial hemorrhage and pain were recorded [30, 31]. Here we reported three cases with lung tumors treated by PLA with fine needle and lower energy. We successfully applied CT-guided laser ablation into the lesions in the lung, and postoperative follow-up showed good results.

## CASE PRESENTATION

We retrospectively collected three patients who underwent CT-guided PLA for liver metastatic lung cancer from January 2016 to March 2016 at The First Affiliated Hospital, College of Medicine, Zhejiang University. We targeted these three patients who were evaluated according to the CT imaging. Surgery and chemotherapy were carried out in two patients due to sigmoid colon metastases. The study was approved by the ethics committee of The First Affiliated Hospital of Zhejiang University. These patients had written informed consent for the use of their clinical data and tissues sample for study. Case 1 was a 62-year-old man, who underwent surgical resection of the caudate lobe in March 2013, and two years later, he had TACE in our hospital. Preoperative CT image revealed three lung lesions of 15.4*10.7mm, 14.5*7.7mm and 5*5mm, respectively. This patient could not accepted surgical resection, RFA, MWA or particle implantation treatment because of severe pulmonary dysfunction and diffuse pulmonary bullae. Therefore, CT-guided PLA procedure was conducted. Case 2 was a 64-year-old man with only one nidus. He had a history of the sigmoid colon tumor resection in 2011, and gamma knife surgery of liver cancer in 2013. Computed tomography detected a 11.6*7.5mm nodule in the right lung. He also underwent a CT-guided PLA procedure within two laser fibers, each of 1800J. Case 3 was a 63-year-old woman with sigmoid colon metastatic lung cancer, who had the sigmoid colon tumor and liver metastases resection in 2014. CT revealed a 13*9 mm lesion of the right-middle lobe.

## CT-GUIDED PERCUTANEOUS LASER ABLATION

**Figure 1:**
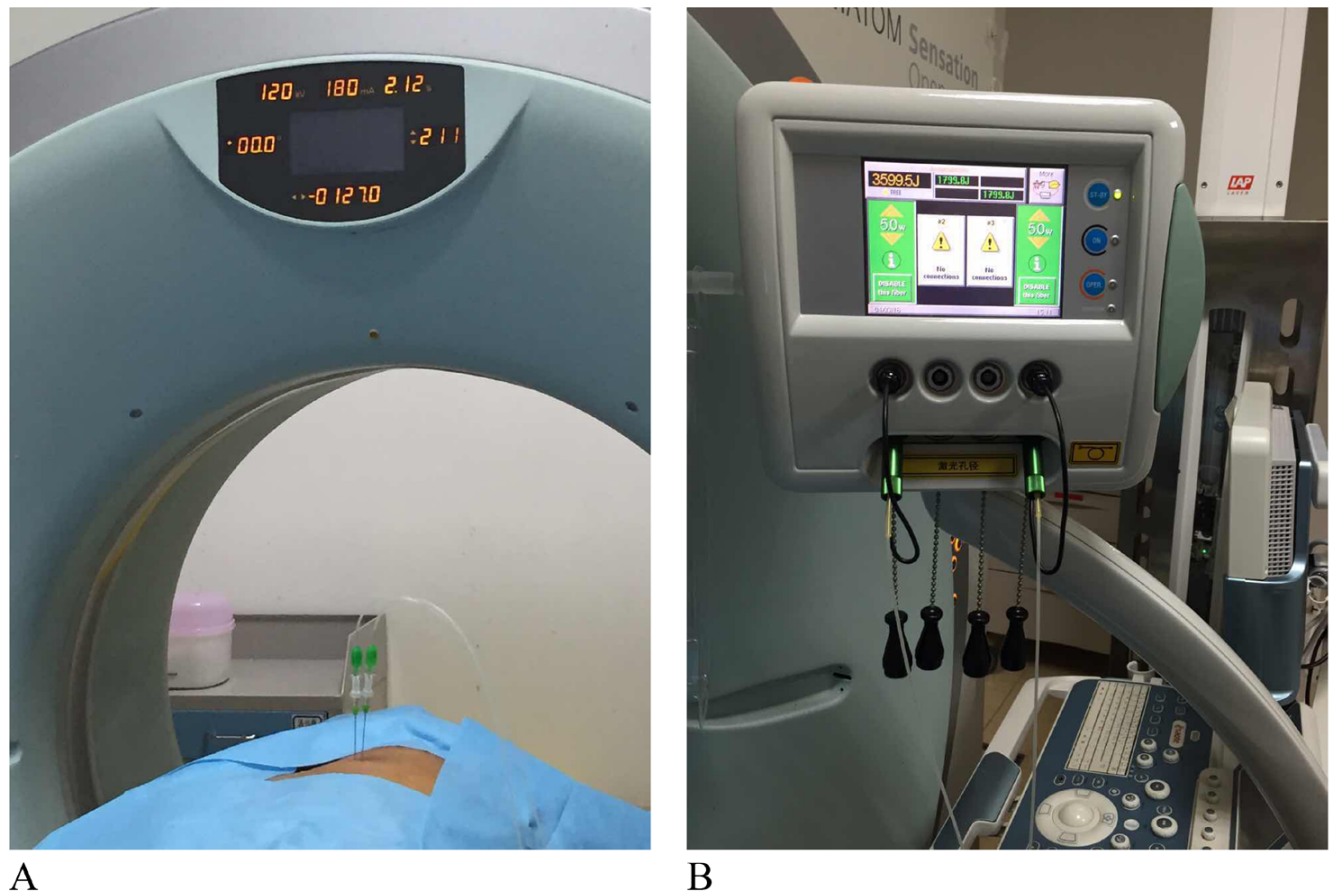
Mechanisms of CT-guided percutaneous laser ablation

The procedures were performed under local anesthesia, in which lidocaine 2% was administered. Computed tomography (CT) was implemented to localize the nidus by 64-slice systems (Brilliance iCT and 64-channel systems). With a 21-gauge Chiba needle (Top, Tokyo, Japan), the interventional radiologist withdrew the needle core and placed the laser fiber into the needle sheath outside of the pin end within 1 cm. If the tumor is larger, we can ablate it simultaneously with at most 4 laser fibers. The nidus was ablated by a Nd:YAG laser fiber with a wavelength of 1064nm, each of which has an output power of 5W for 1800J (Echolaser X4, ESAOTE, Italy) (Figure 1A and 1B). Targeted tumour tissue would be in coagulation necrosis when the tissue temperature reached 55°C. When there was residual area left, an additional laser ablation should be conducted subsequently.

## FOLLOW-UP AND OUTCOME MEASURES

The outcome of the local ablation was evaluated according to the World Health Organization (WHO) response evaluation criteria for solid tumor: 1) Complete response (CR): disappearance of all target niduses without any residual lesion for a month; 2) Partial response (PR): 50% or more reduction in target lesions, without a 25% enlargement in any one target lesion for a month; 3) Stable disease (SD): neither PR nor PD criteria fit; 4) Progressive disease (PD): 25% or more enlargement in target lesions or appearance of new lesions [32]. Patients were observed 24h after the CT-guided PLA for possible complications including fever, skin burns, and pain. Serum biochemical tests, including white blood count (WBC), alpha fetal protein (AFP) levels, carbohydrate antigen 199 (CA199) levels, and carcinoembryonic antigen (CEA) levels (reference range, (4-10) *10E9/L, (0-20) ng/mL, (0-37) U/mL, (0-5) ng/mL, respectively), were obtained before the first PLA session and 1 week after PLA session. In addition, we also conducted a systematic review by retrieving electronic databases of PubMed, Embase and Scoups using the words as following: lung, computed tomography, metastasis, neoplasm, radiofrequency ablation, microwave ablation and laser ablation in recent five years without language limitations (see Appendix 1).

## RESULTS

**Figure 2:**
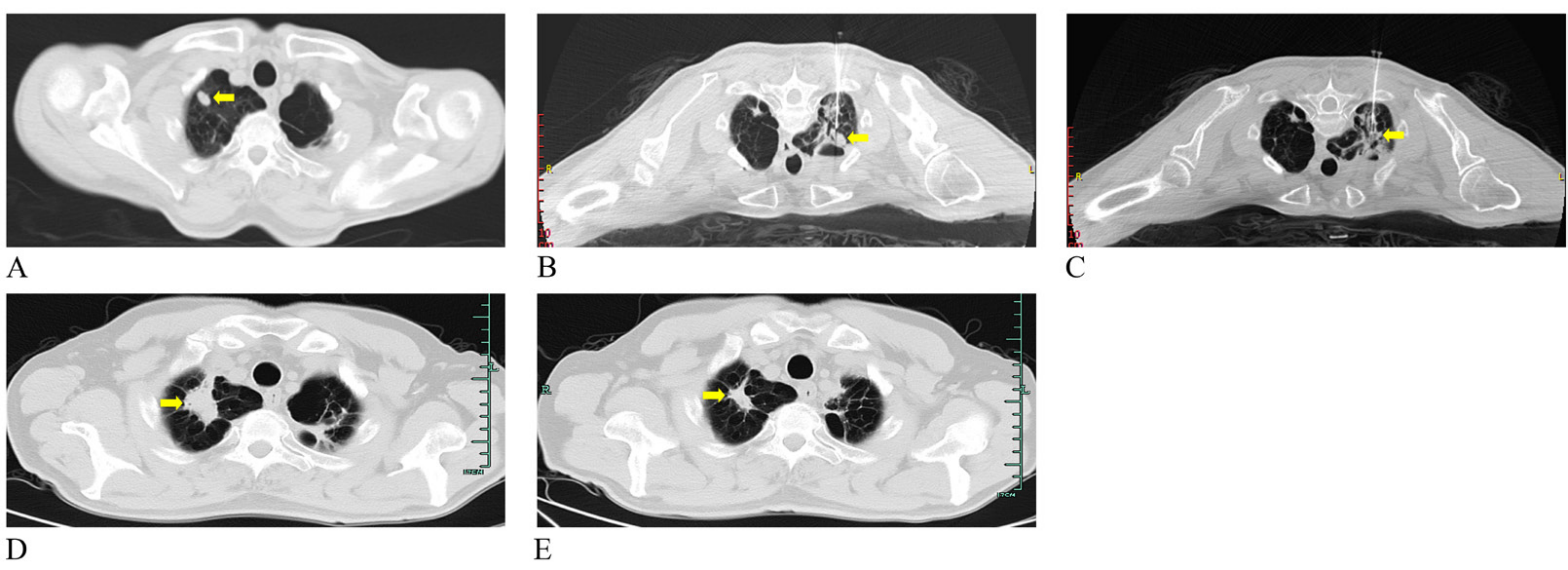
Representative CT images from a 62-year-old man diagnosed with lung metastasis from liver cancer **A.** Preoperative unenhanced CT image before PLA shows tumor about 14.5*7.7 mm in the right lung (yellow arrow). Intraoperative CT image shows the arranging needle method of using two laser fibers parallelly ablating the tumor (**B.** Before ablation; **C.** After ablation) (yellow arrows). Postoperative CT image in axial plane indicated the increased high-density lesion that has been ablated two months later **D.** and reduced in four-month follow-up **E.** (yellow arrows).

**Figure 3:**
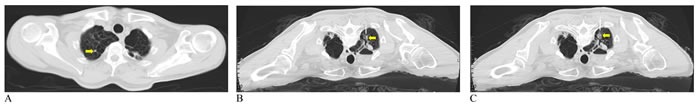
**A.** Preoperative CT image showed a mass in the upper lobe of the right lung (yellow arrow). **B.** A laser fiber was inserted into the location 1 cm away from the lesions (yellow arrow). **C.** CT image showed successfully ablated with high-density signal (yellow arrow).

**Figure 4:**
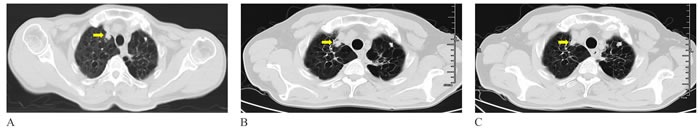
**A.** Supine CT image before PLA showed the tumor measuring 15.4*10.7 mm in diameter adjacent to the blood vessels in the right lung (yellow arrow). **B.** and **C.** CT image two and four month obtained after ablation showed the lesion not significant change (yellow arrows).

**Figure 5:**
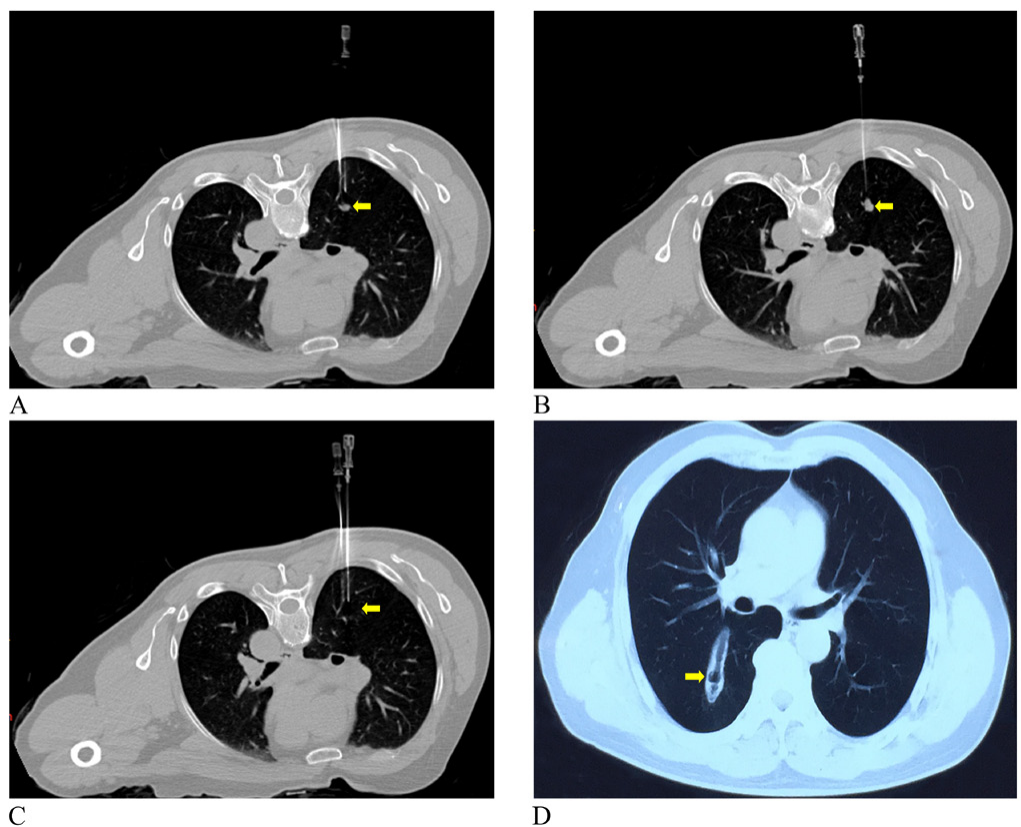
A 64-year-old man with lung metastasis from sigmoid colon cancer Coronal unenhanced CT images revealed laser fibers ablating the tumor 1 cm outside the lesion (**A.** Before ablation; **B.** After ablation) (yellow arrows). **C.** CT image during the two-month follow-up demonstrated completed response (yellow arrow).

**Figure 6:**
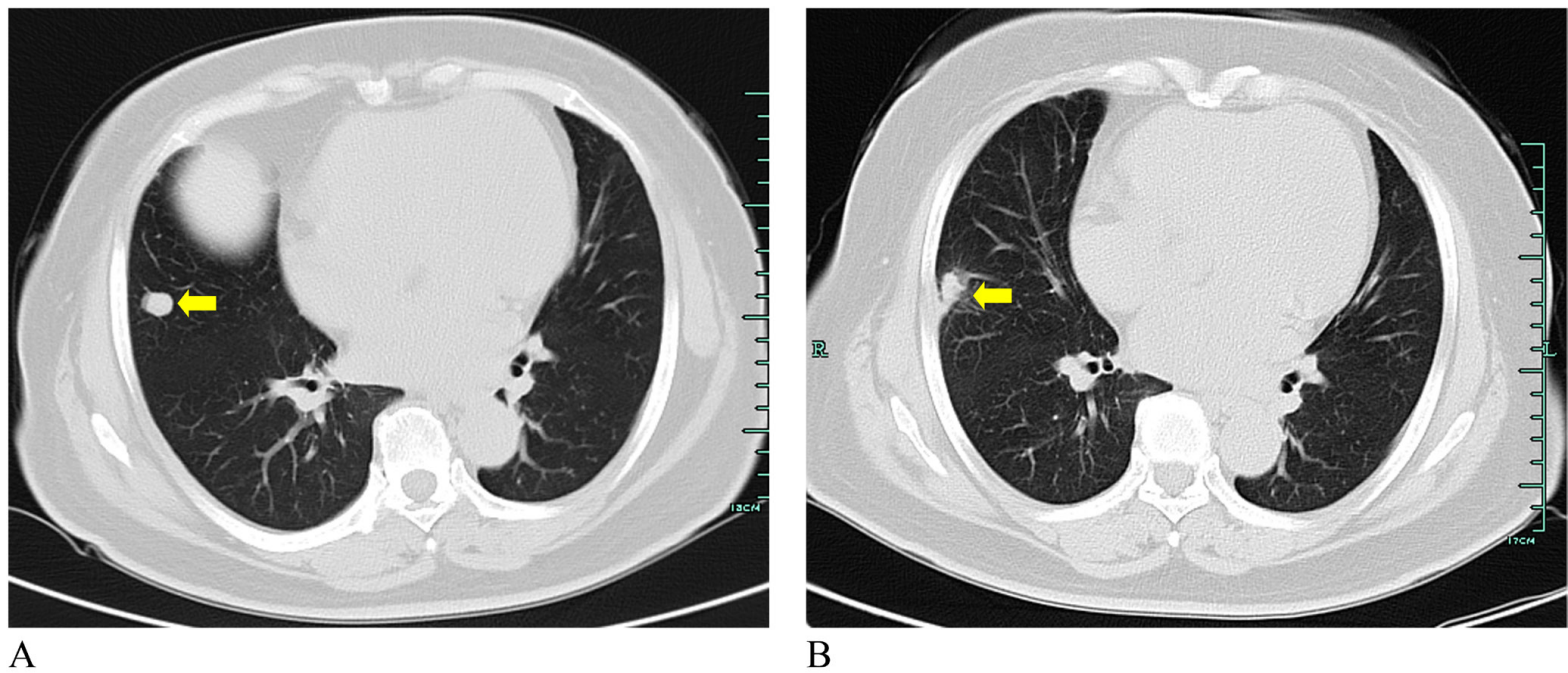
CT images from a 63-year-old woman with lung metastasis from sigmoid colon cancer Coronal unenhanced CT images indicated the decreased tumor after PLA for lung metastasis (**A.** Before ablation; **B.** After ablation) (yellow arrows).

The basic characteristics of three cases are summarized in Table 1. These three patients were aged from 62 to 64 years. The major tumor axes ranged from 5 mm to 15.4 mm. Case 1 mentioned of cough and some dyspnea after the first interventional therapy. Chest radiograph suggested small pneumothorax requiring chest tube drainage. CT scan obtained four months following the ablation showed two lesions with complete response (Figure 2A-2E and Figure 3A-3C) and one partial response (Figure 4A-4C). The WBC, AFP, CA199, and CEA levels in the patient were all normal before and after the laser ablation (Table 1). Case 2 also had a successful complete response with absent lung mass (Figure 5A-5D). Although other blood parameters were within the reference range, the level of CEA was high in both before and after the ablation. However, the patient had a good postoperative condition without discomfort in the two-month follow-up. Case 3 showed partial response and improved greatly after five months (Figure 6A and 6B). Most of the blood biochemical parameters were normal except for CEA level.

**Table 1:** Case and tumor characteristics

Patient	Sex	Age (years)	Tumor size (mm)	Characteristics of patients	Primary pathological type	Prognosis	Follow-up (months)	WBC (10E9/L)		AFP (ng/mL)		CEA (ng/ml)		CA19-9 (U/ml)		Number of fibers	Type of needle used for CT-guided PTA	Power (W)	Energy (J)	Complication/Outcome
								Before	After	Before	After	Before	After	Before	After					
Case 1	Man	62	15.4*10.7	Liver metastatic lung cancer	Hepatocellular carcinoma	PR	Four	4.8	6	1.6	1.2	3.7	2.2	<2.0	<2.0U/ml	1	21G, long	5	3600	Pneumothorax/Alive
			14.5*7.7			CR	Four									2	21G, long	5		
			5*5			CR	Four									1	21G, long	5		
Case 2	Man	64	11.6*7.5	Sigmoid colon metastatic lung cancer	Middle differentiated adenocarcinoma	CR	Two	4.6	5.8	1.7	2.2	12.8	6.8	10.8	10.7	2	21G, long	5	3600	Alive
Case 3	Woman	63	CT 13*9	Sigmoid colon metastatic lung cancer	Middle differentiated adenocarcinoma	PR	Five	4.5	3.81	1.3	1.36	43.9	44	5.9	5.92	2	21G, long	5	3600	Alive

WBC: white blood count;

AFP: alpha fetal protein;

CA199: carbohydrate antigen 199;

CEA: carcino embryonie antigen;

PLA: percutaneous laser ablation;

PR: partial response;

CR: Complete response.

## DISCUSSION

Lung cancer is an important cause of cancer-related death in the world. Recently, emerging studies have depicted thermal ablation including RFA, MWA, PLA to be applied on the tumors. Our study mainly indicated that CT-guided PLA has significant effectiveness in the lung nidus. The therapy was optimized by using CT to evaluate the ablated area immediately after laser ablation. In addition, the procedure had only transient adverse effects without serious complications.

We reviewed 27 studies presenting a total of 962 cases with 1297 lung tumors following percutaneous CT-guided RFA, MWA and LA. The summary of 27 studies is listed in Table 2 [5-15, 17-22, 30, 31, 33-40]. The mean age of included studies was beyond 45.7 years, and 62.2% were males. The majority of the lung tumors were completely or partially ablated. The complication rate occurred in percutaneous CT-guided RFA was 12.8%-24.6% [8, 9] and in MWA was 11.9%-66.7% [5, 7]. Li et al. used CT-guided RFA percutaneously as a supplemental therapy for selected advanced non-small cell lung cancers. 31 patients had complete responses, 12 patients had partial responses, 6 patients stable disease, and no patients progressive disease [5]. However, pneumothorax occurred in 8 of 67 patients [5]. Li et al. successfully performed C-arm CT to RFA for 36 small lung lesions. During the procedure, 4 cases had pneumothorax and 3 cases developed hemoptysis [6]. In another study, 12 patients were successfully treated by RFA via a transosseous route under CT guidance. Several complications including pneumothorax, pulmonary hemorrhage, hemothorax, pneumonia, and fever also appeared [7]. There were also similar postoperative side reactions in CT-guided microwave ablation [8-10]. Previous multivariate analysis found that the prognosis in pulmonary metastatic patients treated by RFA was significantly associated with the size and the number of metastasis [41]. Compared to these thermal ablation in lung nidus, our study suggested that CT-guided PLA manifested fewer side effects and had predictable, small, and legible volumes of necrosis. These may be because PLA has thinner puncture needle, smaller thermal effect and more effective range than other thermal ablations. Laser fibers were used parallelly for larger lesions, and the combination required less energy for focal ablation and gasification. It was reported that when energy input was excessive, it might lead to malignant tumor recurrence and rapid expansion [42-44]. Therefore, in order to inactivate tumor, lower power and energy were used to produce better prognosis. Regarding the process of laser ablation in tissue, it is divided into five stages: first, tissue protein degeneration following laser-induced high temperature; second, coagulation necrosis; third, tissue liquefaction; fourth, moisture evaporation; finally, carbonizing the diseased regions. Interestingly, even if the first needle was deviating from the lesion center, we can place the second needle parallelly to ablate the whole lesions under CT guidance. Preoperative lesions showed high density imaging while it disappeared completely with low density imaging after gasification and ablation. We called this phenomenon “Lesions gasification signs”. This is different from the image of laser ablation in liver lesions.

**Table 2:** Summary of 962 cases with 1297 pulmonary tumors after ablation in 27 published literatures

Author	Year	Country	Characteristics of patients	Treatment method	Patients (No. of tumors)	Tumor size(mm)	Male/female	Mean age (range)	Follow-up interval (months)	Prognosis	Complication
Vogl TJ et al.	2004	Germany	24 lung metastases; 6 localized lung tumors	22 percutaneous CT-guided LA; 8 percutaneous MR-guided LA	30(30)	0-30	14/16	60.2 (35-81)	36	24 CR	3 pneumothorax
Schoellnast H et al.	2011	America	1 NSCLC	Percutaneous real-time FDG PET CT-guided RFA	1(2)	18*12; 16*8	1	75	12	PR	NA
Vogl TJ et al.	2011	Germany	80 lung metastases	Percutaneous CT-guided MWA	80(130)	2.9±2.4 mL	30/50	59.7±6.4 (48-68)	6-24	Technical success rate: 73.1%;1- and 2-year OS: 91.3% and 75%	11 pneumothorax;8 hemorrhage; 6 hemoptysis; 12 pain;1 burn
Schoellnast H et al.	2012	America	26 adenocarcinoma; 9 squamous cell carcinoma; 1 large cell carcinoma; 3 NSCLC	Percutaneous CT-guided RFA	33(39)	28±15 (10-75)	21/12	75±7	19±11 (1-52)	Technical success rate: 97%;a)<3cm median OS: 24 months;1-year OS rate: 83%;median PFS: 11 months;1-year PFS rate: 50%;median TTLP: 24 months;1-year LP free rate: 65%; b)>3cm median OS: 15 months; 1-year OS rate: 56%;median PFS: 5 months;1-year PFS rate: 11%;median TTLP:8 months;1-year LP free rate: 17%	1 died;10 pneumothorax; 1 brachial plexopathy
Okuma T et al.	2012	Japan	1 NSCLC complicated with mild interstitial pneumonia	Percutaneous CT-guided RFA	1	23	1	67	11	CR	1 pneumothorax;1 pleural effusion
Lu Q et al.	2012	China	48 NSCLC; 21 pulmonary metastasistumor	Percutaneous CT-guided MWA	69(93)	22.3±1.7 (8-55 )	45/24	65±15	36	a)NSCLC: 1-, 2- and 3-year OS:75.0%, 54.2% and 29.2%; 1-, 2- and 3-year recurrence-free survival rates:72.9%, 50.0% and 27.1%; b)pulmonary metastatic tumor: 1-, 2- and 3-year OS:47.6%, 23.8% and 14.3%;1-, 2- and 3-year mortality rates for pulmonary metastatic tumor patients: 47.6%, 19.0% and 14.3%	13 pneumothorax;5 hemoptysis;2 hemothorax;3 pneumonia; 2 pain;2 fever
Liu H et al.	2013	Australia	5 adenocarcinoma; 6 NSCLC; 4 squamous; 1 large cell	Percutaneous CT-guided MWA	15(16)	24	11/4	73 (52-87)	12 (6-18)	9 CR; 2 PR; 5 LTP	10 pneumothorax;1 haemoptysis
Belfiore G et al.	2013	Italy	44 lung cancer, 25 lung metastases	Percutaneous CT-guided MWA	56(69)	30±9	35/21	61.5±9.13	3-36	Mean OS:27.8±2.8 months;1-, 2- and 3-year cancer-specific mortality: 69%, 54% and 49%	18 pneumothorax; 8 pain
Li X et al.	2013	China	20 squamous cell carcinoma; 26 adenocarcinoma; 3 others	Percutaneous CT-guided RFA	49(61)	29 (14-50)	40/9	Median: 60 (24-82)	19 (6-34)	31 CR; 12 PR; 6 stable status;median PFS: 16 weeks	Major complication: 8 pneumothorax; minor complications: self-limited minor pneumothorax; slight cough, fever; local pain
Gadaleta CD et al.	2013	Italy	1 squamous cell carcinoma; 2 NSCLC; 17 metastatic lung cancer	Percutaneous CT-guided RFA	17(20)	32±7	6/11	66.5 (44-81)	6	11 CR;technical success rate: 100%;LTP rate: 21% in 3-5-cm-diameter tumors and 0% in tumors of 3 cm or smaller in diameter	Major complications: 5 pneumothorax; 1 bronchopleural fistula
Yang X et al.	2014	China	28 adenocarcinoma; 13 squamous; 6 others	Percutaneous CT-guided MWA	47	24-50	30/17	69.4 (56-82)	30	Median time of the first recurrence,cancer-specific and median OS: 45.5, 47.4 and 33.8 months;after MWA 1-, 2-, 3- and 5-year OS rate: 89%, 63%, 43% and 16%;after MWA 1-, 3-, 5-year local control rate: 96%, 64% and 48%	Major complications: 5 pneumothorax;3 pleural effusion;1 bronchopleural fistula; minor complications: 15 pneumothorax;7 pleural effusion;10 hemoptysis;7 pneumonia
Li XQ et al.	2014	China	36 small lung lesions	Percutaneous C-arm CT-guided RFA	34(36)	18±7 (4-30)	20/14	56±3 (28-80)	16.5±13.1 (2-56)	6-month, 1- and 2-year OS:100.0,69.0 and 60.0%	4pneumothorax; 3 hemoptysis
Hu X et al.	2014	China	8 primary lung cancer; 6 adenocarcinoma/bronchioloalveolar; 2 squamous-cell cancer; 10 pulmonary metastasis from HCC	Percutaneous CT-guided RFA	26(29)	20±4 (10-35)	19/7	61.3±6.7 (44-77)	13.5 (3-30)	Technique efficacy rates in primary tumors and lung metastases patients: 93% and 91.7%;2-year OS rate: 80.8%;2-year-cancer-specific survival rate: 100%;1- and 2-year-tumor-free survival: 69.2% and 26.9%	5 pneumothorax;3 hemoptysis
Nour-Eldin NE et al.	2014	Germany	6 primary lesions; 94 metastatic lesions	Percutaneous CT-guided RFA	78(100)	NA	46/32	58.9	12	80 CR;mean detectable time of tumor residue or recurrence after ablation: 6.7 months	8 pneumothorax;6 hemorrhage;4 hemoptysis
Iguchi T et al.	2015	Japan	12 lung tumors	Percutaneous CT-guided RFA	12(12)	10±3 (4-16)	6/6	66.4±8.6 (56-81)	19.5 (3.0-41.5)	6-month, 1- and 2-year technique efficacy rate: 91.7%, 81.5%, and 81.5%	3 pneumothorax;1 neuralgia;1 pulmonaryhemorrhage;1 hemothorax;1 andpneumonia;1 fever
Liu L et al.	2015	China	lung cancer	Percutaneous CT-guided RFA	30(30)	13.25±3.15 cm2	19/11	69 (52-85)	NA	CR or PR	NA
Splatt AM et al.	2015	Australia	44 NSCLC; 26 lung metastasis	Percutaneous CT-guided MWA	51(70)	24.4 (7-63)	33/18	71.2 (46-88)	48	NA	Major complications:1 died;9 pneumothoraces;4 effusion drainage;2 pulmonary haemorrhage; 2 infections;1 mechanical failure;1 chest wall burn;1 pleural seeding
Sun YH et al.	2015	China	15 primary lung cancer; 14 metastatic lung cancer	Percutaneous CT-guided MWA	29(39)	37 (15-58)	17/12	Median: 63 (39-74)	Median: 25 (3-45)	8 CR; 14 PR; 4 stable status, and 3 LTP;Mean PFS: 14.6 months; 1- and 2-year OS: 91.3% and 82.6%	5 pneumothorax; 2 pleural effusion; 15 fever
Ni X et al.	2015	China	25 NSCLC; 10 squamous cell carcinoma	Percutaneous CT-guided MWA	35(39)	30 (10-110)	25/10	59 (34-71)	17.7 (6-45)	32 CR; 7 PR;local efficacy: 87.2%; Median MWA-related PFS, MWA-related OS, PFS, and OS:5.4, 10.6, 11.8 and 17.7 months	8 pneumothorax;6 pleural effusion;2 pneumonia;1 bronchial fistula;3 hemorrhage;9 pain
Qi H et al.	2015	China	19 right lung metastases from nasopharyngeal carcinoma and 10 left lung metastases	Percutaneous CT-guided MWA	17(29)	8-42	15/2	45.7 (28-65)	14 (3-24)	6 new metastases	2 pneumothorax;4 parenchyma bleeding
Tavares E Castro A et al.	2015	Portugal	20 primary lung cancer; 8 metastatic lung cancer	Percutaneous CT-guided RFA	28(28)	30.0±13.6 (9-70 )	22/6	62±17	24	Technical success rate: 74.1%;mean OS:43 months;median PFS: 31.6 months;Disease-related mortality: 46.4	7 pneumothorax;1 pleural effusion;3 mildbleeding;1 hemoptoic sputum;2 haemorrhages
Acksteiner C et al.	2015	Australia	11 NSCLC	Percutaneous CT-guided MWA	10(11)	19 (10-52)	6/5	79 (75-88)	12	3 CR;5 PR	2 pneumothorax;chest tube 3;1 chest wall burn;1 chest wall burn;infection 2;1 haemoptysis;5 alveolar haemorrhage;2 surgical emphysema
Cheng M et al.	2016	Australia	recurrent NSCLC	Percutaneous CT-guided RFA and MWA	12(12)	34.2±12.8	8/4	71±7	19±11	Median OS: 35 months;mean time to local progressionfor <30mm and >30mm tumours: 23 months and 14 months	5 pneumothorax
Parvizi N et al.	2016	UK	13 primary lung cancer; 21 metastatic lung cancer	Percutaneous CT-guided MWA	31(34)	19 (10-52)	19/12	72.7 (48-90)	12	7 LTP	NA
Fanucchi O et al.	2016	Italy	25 right upper lobe;3 middle lobe;20 right lower lobe;23 left upper lobe;28 left lower lobe	Percutaneous CT-guided RFA	61(86)	20	38/23	74	28	1-, 3-, 5-year OS rates:94.8% , 49.0% and 44.5%;1-, 3-, 5-year PFS rates were 86.3%, 70.3% and 68.3%	9 pneumothorax;1 chest drainage;2 pleural effusion
Vogl TJ et al.	2016	Germany	231 colorectal lung metastases	a)Percutaneous CT-guided MWA; b)percutaneous CT-guided LA; c)percutaneous CT-guided RFA	109(231)	a)5-50; b)10-45; c)8-42	a)29/18; b)14/7; c)28/13	a)64.6±11.5 (34–86); b)72.9±10.4 (54–94); c)71±10 (50–90)	24	a)The median time to LTP:7.5 months;median survival:32.8 months;1-, 2-, 4-year OS rates:82.7%, 67.5% and 16.6%;1-,2-,3- and 4-year PFS rate:54.6%, 29.1%, 10.0% and 1.0%;b)the median time to LTP:10.4 months;median survival:22.1 months;1-, 2-, 4-year OS rates:95.2%, 47.6% and 23.8%;1-,2-,3- and 4-year PFS rate:96.8%, 52.7%, 24.0% and 19.1%;c)the median time to LTP:7.2 months;median survival:24.2 months;1-, 2-, 4-year OS rates:76.9%, 50.8% and 8.0%;1-,2-,3- and 4-year PFS rate:77.3%, 50.2%, 30.8% and 16.4%	a)14 pleural effusion;1 subcutaneous emphysema;5 hemorrhage;5 hemoptysis;3 pneumothorax;b)22 pleural effusion;6 subcutaneous emphysema;7 hemorrhage;5 hemoptysis;13 pneumothorax;c)37 pleural effusion;31 subcutaneous emphysema;10 hemorrhage;5 hemoptysis;37 pneumothorax
Crombé A et al.	2016	France	1 lung metastase	Percutaneous CT-guided RFA	1(3)	7-12	1	82	120	CR	1 chest drainage

NSCLC: non-small cell lung cancer;

RFA: radiofrequency ablation;

MWA: microwave ablation;

CT: computed tomography;

PR: partial response;

CR: Complete response;

NA: not available;

OS: overall survival;

PFS: progression-free survival;

TTLP: time to local progression;

LTP: local tumor progression.

Nevertheless, there are several limitations of CT guidance:1) Imaging plane orientation for needle position is strict. 2) A small gantry bore restricts the needle length. 3) It can not be visualised in real time [45]. In addition, the result may produce bias due to the limited sample size and period of follow-up.

In a word, this study indicated that CT-guided PLA is a minimally invasive, safe and effective method and could be promising for the treatment of lung tumors. Furthermore, especially multineedle PLA in large tumors, there are still the needs for more large-scale prospective studies to convince this in the future.

## SUPPLEMENTARY MATERIALS FIGURES


