# Supplementary material for: CT-guided percutaneous laser ablation of metastatic lung cancer: three cases report and literature review
Source: Oncotarget. 2016 Dec 10;8(2):2187–96. doi: 10.18632/oncotarget.13901 (PMC5356791; doi:10.18632/oncotarget.13901)

CT-guided percutaneous laser ablation of metastatic lung cancer: three cases report and literature review

Supplementary Material

Search strategy:

Pubmed (1950-present)

1. (lung OR pulmonary)
2. ("CT" OR "Computed Tomography")
3. (metastasis OR metastatic)
4. (neoplasm OR tumor OR tumour OR cancer OR oncolog* OR carcinoma)
5. ("RFA" OR "radiofrequency ablation" OR "RF ablation" OR "radiofrequency thermal ablation" OR "RTA")
6. ("microwave ablation" OR "microwave thermal ablation" OR MWA)
7. (laser AND ablation)
8. 5 OR 6 OR 7
9. 1 AND 2 AND 3 AND 4 AND 8
10. "Ablation Techniques"[Mesh]
11. " Laser Therapy "[Mesh]
12. 10 OR 11
13. " Lung Neoplasms "[Mesh]
14. " Carcinoma, Non-Small-Cell Lung"[Mesh]
15. " Small Cell Lung Carcinoma"[Mesh]
16. 13 OR 14 OR 15
17. 12 AND 16
18. 9 OR 17 Sort by: PublicationDate Filters: published in the last 5 years

Embase(1980-present)

1. ' lung':ab,ti
2. ' pulmonary ':ab,ti
3. 1 OR 2
4. ' CT ':ab,ti
5. ' Computed Tomography ':ab,ti
6. 3 OR 4
7. 'metastasis':ab,ti
8. 'metastatic':ab,ti
9. 7 OR 8
10. ' microwave ablation ':ab,ti
11. ' microwave thermal ablation ':ab,ti
12. ' MWA ':ab,ti
13. 10 OR 11 OR 12
14. ' RFA ':ab,ti
15. ' radiofrequency ablation ':ab,ti

16. 'RF ablation':ab,ti
17. 'radiofrequency thermal ablation':ab,ti
18. 'RTA':ab,ti
19. 14 OR 15 OR 16 OR 17 OR 18
20. 'laser ablation':ab,ti
21. ' neoplas*':ab,ti
22. ' cancer ':ab,ti
23. ' tumor ':ab,ti
24. ' tumour ':ab,ti
25. ' carcinoma ':ab,ti
26. ' oncolog*':ab,ti
27. 21 OR 22 OR 23 OR 24 OR 25 OR 26
28. 13 OR 19 OR 20
29. 3 AND 6 AND 9 AND 27 AND 28 Sort by: PublicationDate Filters: published in the last 5 years

Scoups

1. TITLE-ABS-KEY (“lung”)
2. TITLE-ABS-KEY (“pulmonary”)
3. 1 OR 2
4. TITLE-ABS-KEY ("RFA")
5. TITLE-ABS-KEY (“radiofrequency ablation”)
6. TITLE-ABS-KEY ("RF ablation")
7. TITLE-ABS-KEY ("radiofrequency thermal ablation")
8. TITLE-ABS-KEY ("RTA")
9. 4 OR 5 OR 6 OR 7 OR8
10. TITLE-ABS-KEY ("microwave ablation")
11. TITLE-ABS-KEY ("microwave thermal ablation ")
12. TITLE-ABS-KEY ("MWA")
13. 10 OR 11 OR 12
14. TITLE-ABS-KEY ("laser ablation ")
15. 9 OR 13 OR 14
16. TITLE-ABS-KEY ("neoplas*")
17. TITLE-ABS-KEY ("cancer")
18. TITLE-ABS-KEY ("tumor")
19. TITLE-ABS-KEY ("tumour")
20. TITLE-ABS-KEY ("carcinoma")
21. TITLE-ABS-KEY ("oncolog*")
22. 16 OR 17 OR 18 OR 19 OR 20 OR 21
23. TITLE-ABS-KEY ("CT")
24. TITLE-ABS-KEY ("Computed Tomography")
25. 23 OR 24
26. TITLE-ABS-KEY ("metastasis ")
27. TITLE-ABS-KEY ("metastatic ")

28. 26 OR 27
29. 3 AND 9 AND 13 AND 15 AND 22 AND 25 AND 28 Sort by: PublicationDate Filters: published in the last 5 years

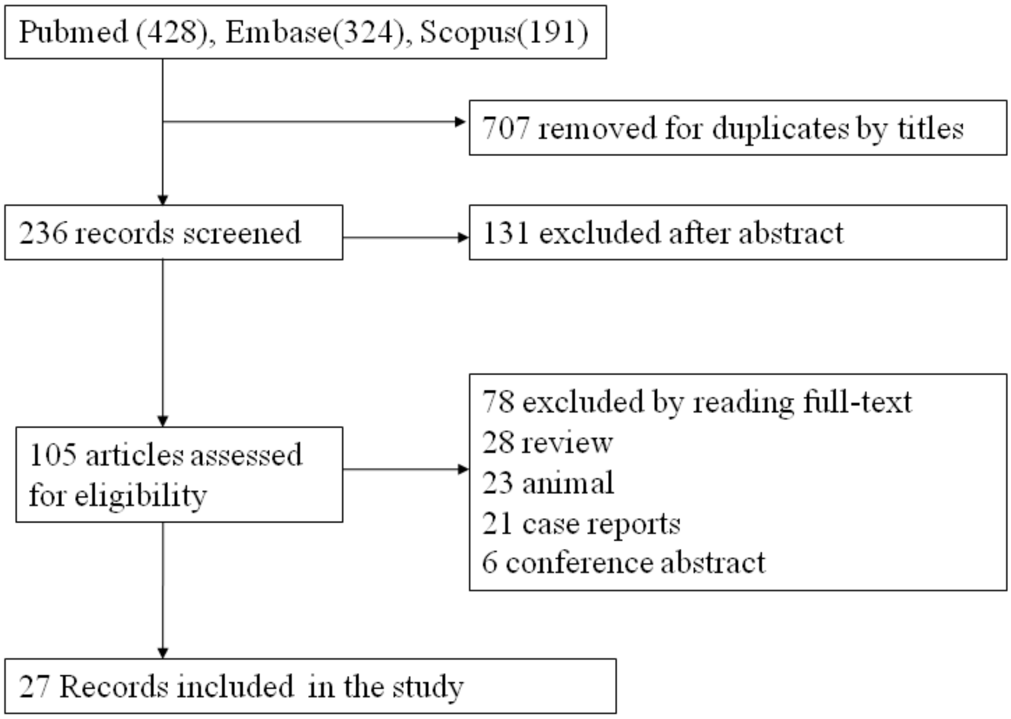

Supplement: Supplementary file 1 [file oncotarget-08-2187-s001.pdf]
